# Estimation of health-related and economic impacts of PM_2.5_ in Arak, Iran, using BenMAP-CE

**DOI:** 10.1371/journal.pone.0295676

**Published:** 2023-12-21

**Authors:** Maryam Salehi, Amir Almasi Hashiani, Behrooz Karimi, Seyed Hamed Mirhoseini

**Affiliations:** 1 Department of Environmental Health Engineering, School of Health, Arak University of Medical Sciences, Arak, Iran; 2 Department of Epidemiology, School of Health, Arak University of Medical Sciences, Arak, Iran; Kanazawa University: Kanazawa Daigaku, JAPAN

## Abstract

Ambient air quality is one of the most critical threats to human health. In this study, the health and economic benefits of reducing PM_2.5_ were estimated in the city of Arak during the period of 2017–2019. The concentration data were obtained from the Environmental Protection Organization of Central Province, while the demographic data were obtained from the website of the Iran Statistics Center. The number of premature deaths from all causes, ischemic heart disease, chronic obstructive pulmonary disease, and lung cancer, attributable to PM_2.5_ pollution was estimated using the Environmental Benefits Mapping and Analysis Program-Comprehensive Version (BenMAP_CE) to limit the guidelines of the World Health Organization. The results showed that improving air quality in 2017, 2018, and 2019 in Arak could prevent the deaths of 729, 654, and 460 people, respectively. The number of years of life lost (YLL) in 2017, 2018, and 2019 was 11383, 10362, and 7260 years, respectively. The total annual economic benefits of reducing the PM2.5 concentration in Arak under the proposed scenarios in 2017, 2018, and 2019 were estimated to be 309,225,507, 262,868,727, and 182,224,053 USD, respectively, using the statistical life method (VSL). Based on the results of this study, there are significant health and economic benefits to reducing PM_2.5_ concentrations in Arak City. Therefore, planning and adopting control policies to reduce air pollution in this city are necessary.

## Introduction

Air pollution is a serious threat to public health and is among the major environmental problems in the current century, especially in developing countries [1, 2]. Research has shown a close relationship between the elevated concentrations of air pollutants and the decreased level of health and well-being of communities, increased mortality rate, reduced field of vision, increased damage to other creatures, and other environmental issues [3, 4]. Air pollution is also related to the ever-growing population, the advancement of industries and technology, the economic status of people, special atmospheric conditions, topography, tobacco, and traffic [5–7]. According to a report published by the World Health Organization (WHO), air pollution causes more than 7 million deaths annually, 90% of which occur in developing countries. This mortality is specifically related to asthma, bronchitis, dyspnea, heart attacks, and various respiratory allergies [8]. Epidemiological studies have demonstrated the annual death of over 800,000 people in the world associated with cardiovascular and respiratory diseases caused by air pollution [9]. Therefore, the determination of the effects of air pollution on public health has been considered.

Although air pollutants enter the human body through the respiratory tract and affect the lungs the most, they can influence other organs of the body as well [10]. According to a report published by the International Agency for Research on Cancer (IARC) in 2013, air pollution and particulate matters (PM) in outdoor air pollution are classified as Group 1 human carcinogens for lung cancer [7]. Among air pollutants, particles smaller than 2.5 μm in aerodynamic diameter (PM_2.5_) have the most adverse effects on human health. This effect is due to their penetration deep into the lungs and reaching other body organs through the circulatory system, thereby causing severe health damage [7, 11].

Air pollution has a variety of social and economic consequences. According to statistics, the economic cost of air pollution in Iran totals 8 billion USD annually, of which 1.5 billion USD is associated with PM [10]. Above these major economic costs, there are alarming statistics on health and hygiene indicators. Statistics show that the health of citizens is in serious danger during polluted days (unhealthy for sensitive groups, unhealthy, very unhealthy, and hazardous conditions) [9].

A variety of applications have been developed to assess the health-related and economic impacts associated with air pollutants. BENMAP is one such tool published by the US Environmental Protection Agency (EPA) in 2003 to estimate the economic benefit of attaining current and potentially future National Ambient Air Quality Standards (NAAQS) [12]. The successful and efficient implementation of management programs to reduce air pollution in cities requires an accurate and precise source of information about the city’s air condition and its impact on human health. The degree to which the people in a community are affected by air pollutants can be determined by estimating the impacts of air pollution. Arak, as the center of Markazi Province (Iran), is located in the passage of trucks and transit vehicles. Its geographical location, linking the south, center, and north of Iran, is the very reason explaining the large number of industries established in this city. Consequently, Arak is one of the most polluted metropolises in Iran [13]. The annual mean concentration of PM_2.5_ in this city is relatively high and its population is exposed to concentrations exceeding the WHO Air Quality Guideline [14]. These factors necessitate taking into account the issue of air pollution and the health of citizens in this city [14]. Although various studies have been conducted on human effects and related costs of air pollution in other cities of Iran, according to the knowledge of the authors, no study has been reported in this field in Arak.

Therefore, this cross-sectional study aimed to estimate the health impacts and economic costs associated with PM_2.5_ in the air of Arak City over a 3-year period using BenMAP-CE software.

## Material and methods

### Study area

Arak, the center of Markazi Province, is located at an altitude of 1708 m above sea level on a wide plain in the neighborhood of the Zagros Mountains (except for the north and northeast). Its longitude and latitude are 49°42′E and 34°06′N, respectively [14]. As the city is situated in an arid and semi-arid region very close to important centers of wind erosion (e.g., a 25,000-hectare desert wetland of Meighan 15 km north-east of Arak), it faces the problem of dust and air pollution and is currently one of the 9 most polluted cities in Iran [13].

### Required data

This study assessed the health-related and economic impacts of reducing the concentrations of particles less than 2.5 μm in diameter up to the limit defined in the WHO air quality guideline in the city of Arak, Iran, using BenMAP-CE v 1.5.8 for the over 30-year-old population over the 2017–2019 period. To estimate the health impacts, this software requires data such as 1) PM_2.5_ concentration over the study years, 2) At-risk population, 3) Basic incidence rate of mortality, 4) Relative risk (RR), and 5) Concentration-Response Functions (CRFs). The desired data for the years 2017 to 2019 were accessed on 10/07/2021.

### Ethics statement

This study has been approved by the Ethics Committee of Arak University of Medical Science with ID: IR. ARAKMU.REC.1400.047.

### PM_2.5_ concentration data

The urban area of Arak has 4 air quality monitoring stations (AQMSs) to measure PM_2.5_. One of the stations provided insufficient reliable data and, thus, was excluded from the study; therefore, only the concentration data of 3 stations were used. The stations of the Environmental Protection Organization were used for the years 2017, 2018, and 2019. The concentration data were processed such that the zero and negative concentrations of all of the stations were removed. Concentrations greater than 3 times the third quartile (75% quartile) were considered outliers and thus removed. Only stations with hourly data of more than 75% during one year (according to WHO criteria) were considered [15, 16]. As there were no reliable data in 2017, only 2 stations were used (Table 1).

**Table 1:** PM_2.5_ distribution in stations and Arak population during the study period.

Year	Number of station	Hourly data coverage	Mean (μg.m^-3^)	Max (μg.m^-3^)	Min (μg.m^-3^)	Total population	Exposed pop. (30–99 years old)
2017	2	77.53%	27.77	76.40	6.63	535,546	293,628
2018	3	78.08%	25.77	73.36	2.43	539,419	295,801
2019	3	81.82%	20.75	50.48	2.52	543,410	297,990

### At-risk population

Information about the Arak population was obtained from the official site of the Statistical Centre of Iran. The population for 2017, 2018, and 2019 was estimated considering the population growth rate of 0.74% based on the 2016 census using Eq (1):

$$P_{n}=P_{0}{\left(1+r\right)}^{n}$$ (1)

where *P*_*n*_ is the population at the end of the period, *P*_0_ is the population at the beginning of the period, n is the time interval between the beginning and the end of the period in years, and r is the average population growth per year [17]. According to the epidemiological studies used in this study, the at-risk population was defined as the population over 30 years of age. Population statistics are presented in Table 1.

### The basic incidence rate of mortality

The analysis of this study is based on natural causes. The basic incidence rates of mortality were extracted from the Institute for Health Metrics and Evaluation (IHME) website for the years 2017, 2018, and 2019. This institute provides annual estimates by 5-year age groups. To measure the PM_2.5_-related deaths, the mortality rates of adults (those above 30 years) were assessed for different age groups with different causes, including all-cause mortality (All-cause), ischemic heart disease (IHD), chronic obstructive pulmonary disease (COPD), and lung cancer (LC) over the study period. The basic incidence rate for each disease was calculated from the ratio of the number of deaths caused by that disease to the at-risk population. The relative risk and the basic incidence rate of mortality per 100,000 over-30-year-old people from Arak are presented in Table 2.

**Table 2:** Baseline mortality rate and relative risks used in this study in people over 30 years old in Arak.

Health endpoint	Baseline mortality rate (per 100,000 population)			Relative risk (95% CI)	Reference
	2017	2018	2019		
All cause	2524	2517	2543	1.06 (1.04–1.08)	Krewski et al. (2009)
IHD	667	665	667	1.24 (1.19–1.29)	
COPD	95	94	95	1.14 (1.06–1.23)	
LC	54	55	57	1.13 (1.1–1.19)	

• Concentration-response functions (CRFs)

The Environmental Benefits Mapping and Analysis Program-Community Edition (BenMAP-CE) is a tool developed by the EPA to assess the number of avoidable premature deaths and illnesses that could result from improving air quality and its associated economic benefits. The mortality rate attributed to the PM_2.5_ (ΔY) pollutant was calculated using Eq (2) [5, 12]:

$$\Delta Y=BI\;\times POP\times (1-{exp}^{-\beta \times DELTAQ})$$ (2)

where BI is the basic incidence of death or illness, POP is the at-risk population (over 30 years old), β is the risk estimation coefficient, and DELTAQ is the difference between the current pollutant concentration (Baseline scenario), and the target concentration (Control scenario) [2, 18]. It is of note that ΔY was calculated separately for each age group and cause of death.

The risk estimation coefficient is a statistical coefficient obtained from epidemiological studies. It calculates the relationship between a one-unit change in the concentration of air pollutants and the health endpoints [19], which can be calculated from Eq (3):

β=ln(RR)/ΔC (3)

where RR is the relative risk and ΔC is the change in the concentration of the air pollutant under study [20, 21]. It is noteworthy that the relative risk coefficients of one of the valuable epidemiological cohort studies were used in this study for health endpoints (i.e., all-cause, IHD, COPD, and LC) [7].

In addition to premature mortality, years of life lost (YLL) were calculated using Eq (4) [22].

$$YLL=\sum {YLL}_{i}{\;YLL}_{i}={\Delta Y}_{i}\times L_{i}$$ (4)

where YLL_i_ represents the years of life lost due to exposure to the PM_2.5_ pollutant, ΔY_i__i is the avoidable premature death of this pollutant, and L is the remaining life expectancy, all for an age group i. In this study, the life expectancy tables presented on the GBD website were used for Iran [23].

### Economic evaluation

Measures such as the cost of illness (COI), willingness to pay (WTP), and human capital approach (HCA) are typically used to estimate the economic benefit of avoiding premature deaths attributed to air pollution. One of the most practical methods used for this purpose is the value of a statistical life (VSL) method based on WTP. This method multiplies the number of predicted deaths by a locally valid estimate of VSL [10]. Since no national or regional studies have estimated the VSL, the results of studies performed in other countries can be used to estimate the VSL of each country.

Comparing the results of the studies carried out in different countries demonstrated a relationship between the Gross domestic product (GDP) per capita and VSL of each country. Therefore, VSL data from Organization for Economic Co-operation and Development (OECD) member countries can be used to estimate the VSL related to Iran (Eq 5). According to the latest estimates, 3.83 million USDs have been proposed for the OECD countries in terms of purchasing power parity (PPP) [11]. This value for Iran can be calculated as follows:

$${VSL}_{Iran}={VSL}_{OECD}\times {\left(\frac{Y_{Iran}}{Y_{OECD}}\right)}^{b}$$ (5)

where VSL_Iran_ is the level of VSL for Iran, Y is GDP per capita, and b is the income elasticity of the VSL. Additionally, b ranges from 1 to 1.4 for low- and middle-income countries, where a median estimate of 1.2 was used [24].

Assuming the equality of the VSL in Iran and Arak, the number of deaths attributed to PM_2.5_ pollution in Arak can be multiplied by the VSL to achieve an economic estimate of this pollutant by adding 10% to the costs of the disease. The calculations related to the VSL are presented in Table 3 [9].

**Table 3:** VSL calculation for Iran, based on VSL of OECD countries and the ratio of GDP per capita.

Parameters	Value (USD)				Reference
	2017	2018	2019		
VSL_OECD_*	3,832,843	3,832,843	3,832,843	at 2011 market rates, PPP	Narain and Sall (2016)
Y_Iran_	5520	5550	5550	GDP per capita	World Bank website, 2021
Y_OECD_	37,416	39,330	39,842	GDP per capita	World Bank website, 2021
VSL_IRAN_	385,637	365,598	359,967	at 2011 market rates	

*Organization for Economic Co-operation and Development

### Scenarios

The baseline scenario is the annual mean concentration of the PM_2.5_ pollutant from the daily calculated data in 2017, 2018, and 2019. The control scenario for the annual mean concentrations of PM_2.5_ was considered the annual WHO guideline limit for particles smaller than 2.5 μm (10 μg/m3).

## Results

### PM_2.5_ concentration changes

The mean concentrations of PM_2.5_ in 2017, 2018, and 2019 were 27.77, 25.77, and 20.75 μg/m3, respectively. These values exceeded the WHO annual air quality guideline (10 μg⁄m^3^) in all three years and indicated a downward trend from 2017 to 2019.

**Fig 1:**
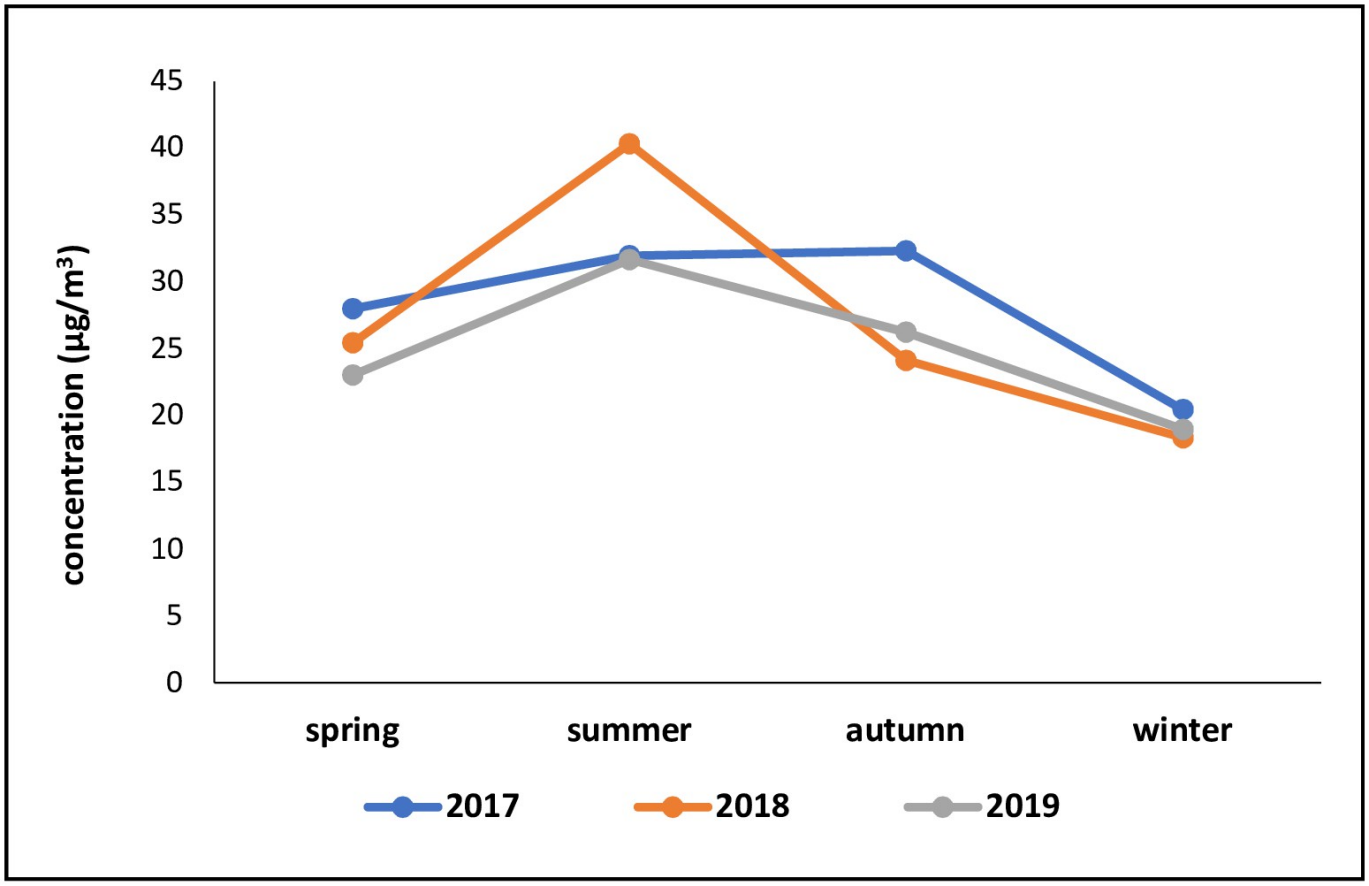
Average pollutant concentration in different seasons in 2017–2019.

Comparing the mean concentration of PM_2.5_ in different seasons of the year shows that the highest concentration level of this pollutant is 40.31 *μg*⁄*m*^3^ in the summer of 2017, and its lowest concentration is 18.33 *μg*⁄*m*^3^ in the winter of 2018 (Fig 1).

### Estimating health-related and economic impacts

#### Mortality associated with PM_2.5_ pollutant.

The long-term impacts of exposure to 2.5-μm particles include the premature death of people due to the failure of vital organs. According to the results of the reduced concentration of particles smaller than 2.5 μm to a concentration of 10 μg/m3 (Table 4), PM_2.5_ is responsible for 9.84% (729 out of 7411 deaths), 31.77% (622 out of 1958 deaths), 19.5% (54 out of 279 deaths), and 20.71% (33 out of 160 deaths) among adults in Arak in 2017 for all-cause, IHD, COPD, and LC, respectively.

**Table 4:** Percentage of deaths attributable to PM_2.5_ among adults in Arak during the study period.

	2017				2018				2019			
	All Cause	IHD*	COPD**	LC***	All Cause	IHD	COPD	LC	All Cause	IHD	COPD	LC
Total mortality	7411	1958	279	160	7445	1967	278	164	7579	2018	284	169
PM_2.5_ mortality	728.96	622.08	54.40	33.15	653.65	565.77	48.79	30.56	460.20	416.62	34.98	22.27
deaths attributable to PM_2.5_	9.84%	31.77%	19.5%	20.71%	8.78%	28.76%	17.55%	18.63%	6.07%	20.64%	12.32%	13.18%

*IHD: Ischemic heart disease,

** COPD: chronic obstructive pulmonary disease,

*** LC: Lung cancer

This pollutant also caused 8.78% (653 out of 7445 deaths), 28.76% (566 out of 1967 deaths), 17.55% (49 out of 278 deaths), and 18.63% (31 out of 164 deaths) among adults in Arak in 2018 for all-cause, IHD, COPD, and LC, respectively.

It also caused 6.07% (460 out of 7579 deaths), 20.64% (416 out of 2018 deaths), 12.32% (35 out of 284 deaths), and 13.18% (22 out of 169 deaths) among the age groups of 30–99 years in Arak in 2019 for all-cause mortality, IHD, COPD, and LC, respectively.

According to the findings, to reach a concentration of 10 μg/m3, 729, 653, and 460 deaths out of the 7411, 7445, and 7579 deaths recorded in 2017, 2018, and 2019 respectively, can be considered avoidable premature deaths attributable to PM_2.5_ in adults over 30 years of age. In other words, 248, 221, and 154 premature deaths per 100,000 adult population of Arak were avoidable in 2017, 2018, and 2019, respectively.

**Fig 2:**
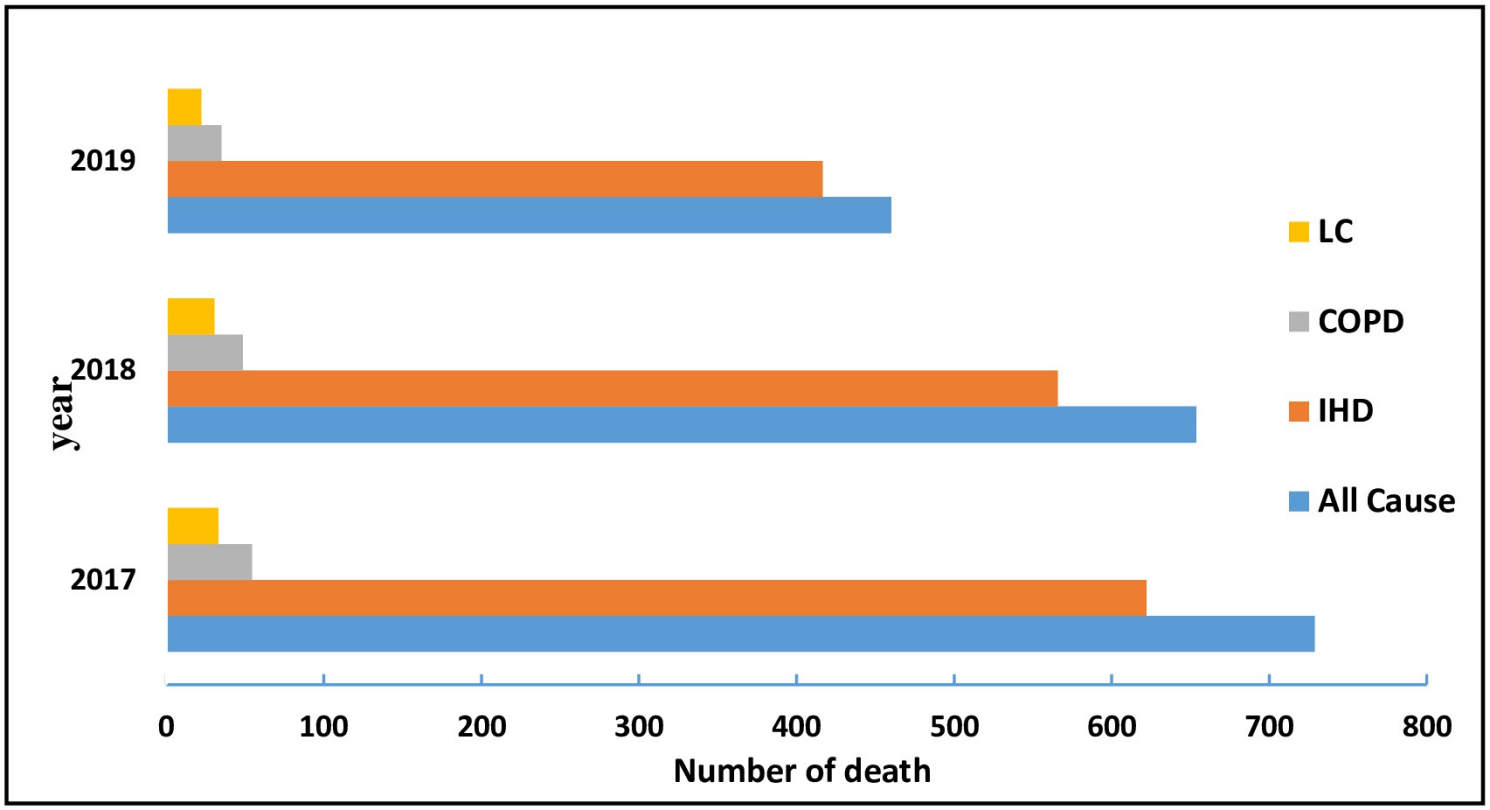
Estimates of avoidable premature deaths attributable to PM_2.5_ stratified by cause of death.

The most common causes of death due to the PM_2.5_ pollutant in Arak are IHD, COPD, and LC, (Fig 2).

#### YLL due to exposure to PM_2.5_ pollutant.

Table 5 shows the number of YLLs due to exposure to the PM_2.5_ pollutant in the outdoor air of Arak in the over 30-year-old population during the study period. According to the results, the average YLLs in this city were 11,384, 10,362, and 7260 in 2017, 2018, and 2019, respectively.

**Table 5:** Estimated age-specific YLL attributable to PM_2.5_ in Arak for 2017–2019, based on IHME life expectancy.

Year	All cause			IHD*			COPD**			LC***		
	Mean	2.5th	97.5th	Mean	2.5th	97.5th	Mean	2.5th	97.5th	Mean	2.5th	97.5th
2017	11,384	7736	14,781	8462	7076	9704	667	531	792	619	277	909
2018	10,362	7028	13,479	7803	6501	8980	632	502	752	579	257	854
2019	7260	4901	9487	5721	4722	6643	451	356	540	422	184	634

*IHD: Ischemic heart disease,

** COPD: chronic obstructive pulmonary disease,

*** LC: Lung cancer

#### Economic calculations

The economic benefit of reducing the PM_2.5_ concentration to 10 μg/m^3^ is presented in Table 6. The results show that the economic benefits from this control scenario in Arak were 309,225,507, 262,868,727, and 182,224,053 USD in 2017, 2018, and 2019, respectively.

**Table 6:** Estimation economic benefits associated with reducing of PM_2.5_ in Arak (USD).

Health endpoint	2017			2018			2019		
	Mean	2.5th	97.5th	Mean	2.5th	97.5th	Mean	2.5th	97.5th
All Cause	309,225,507	210,134,369	401,523,213	262,868,727	178,296,885	341,942,304	182,224,053	123,012,129	238,116,599
IHD*	263,885,960	220,675,678	302,617,482	227,528,565	189,571,493	261,828,821	164,965,195	136,147,222	191,542,976
COPD**	23,075,417	18,362,012	27,405,759	19,623,247	15,575,117	23,362,313	13,850,549	10,922,024	16,592,010
LC***	14,016,092	6,282,277	20,621,834	12,290,749	5,451,776	18,145,855	8,816,316	3,839,542	13,240,307

*IHD: Ischemic heart disease,

** COPD: chronic obstructive pulmonary disease,

*** LC: Lung cancer

## Discussion

Comparing the trend of changes in PM_2.5_ concentration during the study period in the urban area of Arak indicates the unfavorable status of the annual mean concentration of this pollutant in all the studied years compared with the annual WHO standard, 5 μg/m3 (guideline value in 2021), and 10 μg/m3 (guideline value in 2005) [8]. Therefore, detrimental impacts of exposure to PM_2.5_ on the health of the population of this city can be expected. The highest concentration of PM_2.5_ was observed in the autumn of 2017 and in the summer of 2018 and 2019. Air pollution is variable by nature and origin and has significant temporal-spatial variation [25–27]. This phenomenon may be affected during summer by a variety of variables including the size of the city, the industries active in that area, the topography of the city, meteorological parameters, the season, sources of emission of pollutants, and solar radiation due to the presence of dust and PM_2.5_ centers [6, 28]. The increase in PM_2.5_ concentration during the summer may thus be attributed to the presence of dust centers, including that in Meighan Wetland [29]. Therefore, the current study investigated the impact of long-term exposure to the PM_2.5_ pollutant on the health of over-30-year-old people in Arak. The mortality rates in this city per 100,000 people over 30 years of age were 2524, 2517, and 2543 in 2017, 2018, and 2019, respectively. Overall, it was possible to avoid a total of 729, 654, and 460 deaths attributable to PM_2.5_ in our study by applying the control policy of reducing its concentration to 10 μg/m3 in 2017, 2018, and 2019, respectively.

The largest number of deaths occurred in 2017. The PM_2.5_ concentration this year was higher than that in 2018 and 2019. In addition, the findings revealed an increased population in 2019 despite the decreased level of the annual mean concentration of PM_2.5_ compared to that in 2017 and 2018 and the decreased number of resulting premature deaths. These results are consistent with those reported by other related studies, indicating the importance of particle concentration control [30]. Another study in Karaj (Iran) investigated the health impacts of the PM_2.5_ pollutant over the years 2012–2019. The results revealed a larger number of deaths in all years compared to 2013, when there was a lower level of PM_2.5_ concentration [31].

A study in China reported that the number of deaths avoidable from cardiovascular diseases, respiratory diseases, and lung cancer would be 47,000, 89,000, and 32,000, respectively, in 2014 thanks to the dropped level of the PM_2.5_ concentration to 35 μg/m3 (NAAQS) [32]. Additionally, the results of a study conducted in Wuhan (China) showed that premature deaths were avoided by 21,384 due to a 43.6% reduction in PM_2.5_ concentration [16]. Another study in Ho Chi Minh City (Vietnam) indicated that 357, 715, and 64 premature deaths from COPD, IHD, and LC, respectively, would be avoided by reducing the annual PM_2.5_ concentrations from 23 to 10 μg/m3 in 2017 [33]. A study conducted in Cartagena (India) with a control scenario of reducing the baseline concentration by 20% (25 μg/m3) in the over-29-year-old population of the city estimated that control of PM_2.5_ concentration would prevent 104 premature deaths and lead to an economic benefit of 50–100 million USDs [1]. Another study in Tehran (Iran) estimated more than 4000 deaths due to high concentrations of PM_2.5_ with an annual concentration of approximately 31μg/m^3^ [21]. Three control scenarios were used in Ho Chi Minh City to reduce the annual mean concentration of PM_2.5_ (28.9 μg/ m^3^). The β coefficients of different global studies were used in this study. Eventually, by merging the results, the combined number of avoided deaths was 3785, 3195, and 1300, respectively, to reach concentrations of 5, 10, and 25 μg/m^3^ per year in 2019 [34]. In our study, IHD-related deaths were the highest during the study period. These data are consistent with the findings of studies conducted in Qom, Tehran, Karaj, and Isfahan (four Iranian cities) using BenMAP-CE software. In these studies, IHD-related deaths were also more than those with other causes [9, 10, 20, 21, 31]. A study in South Africa showed that reducing the PM_2.5_ concentration to 20 μg/m^3^ (NAAQS) and 10 μg/m^3^ (WHO guideline in 2005) would prevent the premature death of 14,000 and 28,000 people, respectively. Moreover, they showed that implementing this policy would lead to economic benefits of 14 and 29 billion USDs [15].

The calculation of YLL revealed that the concentration of PM_2.5_ is directly related to the number of deaths attributed to this pollutant and the YLL. In the YLL estimation, both the number of premature deaths and the remaining life expectancy for different age groups are considered. According to the results of our study, the largest number of YLLs attributed to PM_2.5_ was 11384 YLLs, which occurred in 2017.

In the present study, the VSL for Iran was calculated based on the VSL of OECD member countries, which was 385,637, 365,598, and 359,967 USDs in 2017, 2018, and 2019, respectively (with a market rate of 2011). The results indicated that the improvement in PM_2.5_ concentration would lead to economic benefits of 309, 263, and 182 million USDs in 2017, 2018, and 2019, respectively. Tinh et al. estimated the economic benefit of reducing PM_2.5_ concentration using two approaches, namely VSL OECD and VSL USEPA, in Ho Chi Minh City, Vietnam. They found that the economic benefit of reducing the concentration of this pollutant to 5, 10, and 25 μg/m^3^ using the OECD VSL approach would be 2.4, 2.1, and 0.8 billion USDs, respectively, while reducing it using the USEPA VSL approach would lead to economic benefits of 3.7, 3.1, and 1.3 billion USDs per year, respectively, in 2019. They concluded that the VSL calculated from the OECD reference could be more suitable for Vietnam [34].

Amoushahi et al. used BenMAP-CE to estimate the PM_2.5_-attributed avoidable health burden and economic loss during 2016–2019 using the CRF of the Global Exposure Mortality Model (GEMM) in Isfahan (Iran). According to their findings, PM_2.5_ -related deaths during the study period were 1,311–1456, and the YLLs resulting from exposure to this pollutant were 22,488–25,614 years. Similar to our study, they used the WTP approach to estimate the avoided annual economic costs associated with PM_2.5_ reduction, which ranged from $526 to $600 million during the study period [9].

Bayat et al. assessed the PM_2.5_-related mortality rate and costs in Tehran for 2017. Using the GEMM function, they estimated the PM_2.5_-attributed deaths for adults to be 7146. According to these authors, about 15% of all deaths originated from exposure to PM_2.5_ pollution. The YLL resulting from exposure to ambient PM_2.5_ in Tehran in that year equaled 109,168 years. Furthermore, the total annual economic benefit resulting from the reduction of PM_2.5_ concentration level to 2.4 μg/m^3^ was 0.591 billion USDs using the Value of a Life Year (VOLY) approach and 2.9 billion USDs using the VSL approach [10].

Safari et al. estimated the health-related and economic effects related to the reduction of the PM_2.5_ pollutant using BenMAP-CE software. They estimated 4694 and 2476 premature deaths under the two control scenarios of reduction to 2.4 and 10 μg/m^3^, respectively. The total YLLs resulting from exposure to PM_2.5_ in scenarios I and II were 158,657 and 78,351 years, respectively. Moreover, the total economic estimate in the two scenarios was calculated as 856 and 451 million USDs, respectively [20].

## Conclusion

The present study showed the impact of the PM_2.5_ pollutant on health and the number of deaths. The results demonstrate that hundreds of deaths could be avoided by improving air quality. For example, reducing the concentration of PM_2.5_ to meet the WHO air quality guideline for an average of three years could help prevent the death of 614 people and the loss of 9669 years and 251 million USDs in Arak. It is, therefore, possible to prevent the abundant health-related and economic costs resulting from air pollution by adopting appropriate control policies and using appropriate applications to control the concentration of PM_2.5_. It is of note that the results of this study are specific to Arak and cannot be generalized to other cities and countries. In this study, the concentrations measured by fixed measuring stations are used as the person’s exposure. In this case, the inhaled concentration may not be the same as the measured concentration, so it is recommended to use mobile devices to measure the concentration in future studies.

## Supporting information

S1 File (RAR)
